# Relative and combined contributions of adverse childhood experiences and self-reported child poverty to health and economic outcomes in adults – a retrospective study in a UK region

**DOI:** 10.1186/s12889-025-23938-z

**Published:** 2025-10-15

**Authors:** Mark A. Bellis, Karen Hughes, Kat Ford, Nadia Butler, Charley Wilson, Zara Quigg

**Affiliations:** 1 Public Health Institute/World Health Organization Collaborating Centre for Violence Prevention, Faculty of Health, Innovation, Technology and Science, Liverpool John Moores University, Liverpool, L2 2ER UK; 2 Policy and International Health, World Health Organization Collaborating Centre on Investment for Health and Well-Being, Public Health Wales, Wrexham, LL13 7YP UK; 3 School of Health Sciences, College of Medicine and Health, Bangor University, Wrexham, LL13 7YP UK

**Keywords:** Adverse childhood experiences, Violence, Poverty

## Abstract

**Background:**

Adverse childhood experiences (ACEs) and childhood poverty (CP) are linked to long-term harms, including poor health, lower educational attainment, and economic instability. However, few studies have examined their independent and combined effects on life course health and prosperity as well as their contributions to intergenerational cycles of poverty and abuse. This study analyses these associations using a large regional survey in northwest England.

**Methods:**

A cross-sectional household survey was conducted across five local authority/administrative areas (November 2023–April 2024) using a stratified quota sample by age, sex and deprivation. Analysis used a sample of 5,330 adults ≥ 18 years old. Along with retrospective questions on nine ACE types and CP measured on a Likert scale (categorised into tertiles), the survey measured self-reported health (chronic health condition, mental well-being and overall health) and self-assessed economic (household income, employment status) outcomes. Statistical analyses included chi-squared tests and logistic regression modelling.

**Results:**

ACE count was significantly related to CP. Increases in category of self-reported CP were associated with 43.7% of 4 + ACEs and 20.5% of 2–3 ACEs reports. ACE count and CP both showed independent dose response relationships with all three health measures. Adjusted prevalence of lower health rose from 20.6% (no ACEs, wealthiest CP tertile) to 45.2% (4 + ACEs, poorest CP tertile). Membership of the poorest CP tertile with no ACEs, or of the wealthiest tertile with 4 + ACEs showed intermediate values (34.6% and 28.9% respectively). Economic outcomes (low income, unemployed/long-term sick) showed similar independent and additive relationships with ACEs and CP. Percentage unemployed/long-term sick rose in the wealthiest CP tertile from 3.8% (0 ACEs) to 5.9% (4 + ACEs) and in the poorest tertile from 8.0% to 12.2% respectively.

**Conclusions:**

Our findings support ACEs being widespread across all socio-economic backgrounds but being more frequent in poorer childhoods. Both ACEs and CP appear independently associated with poorer life course health outcomes with exposure to ACEs adding to the risks from CP. Exposure to ACEs and CP are also associated with poorer economic outcomes in adulthood. Therefore, these childhood experiences in one generation may also increase the risk of CP and ACEs in the children they raise. Targeted interventions and policies are already available that can mitigate the impacts of ACEs and CP, improving health, economic stability and productivity and thus, reducing public service costs and increasing prosperity.

**Supplementary Information:**

The online version contains supplementary material available at 10.1186/s12889-025-23938-z.

## Introduction

Adverse childhood experiences (ACEs) include physical, verbal and sexual abuse of children as well as exposure to domestic violence, substance use and other sources of trauma typically occurring within childhood household environments [1]. Recent discussions have considered the interaction of ACEs with sources of childhood trauma outside of the home such as community violence, school bullying and experience of racial prejudice [2–4]. Socio-economic factors, such as childhood poverty (CP), have also been suggested as important considerations in understanding risk factors for exposure to ACEs and the magnitude of their impact on life course health and well-being [5, 6].

Both ACEs and CP have been linked with similar long-term harms. Multiple studies confirm a dose response relationship between numbers of ACEs and adverse behavioural outcomes in subsequent life stages including greater alcohol, tobacco and drug use along with violence and crime [7–10]. Higher numbers of ACEs also predict increased mental and physical ill health in adulthood, including earlier onset of non-communicable diseases [11–13]. Similarly, growing up in poverty is a key determinant of life course health [14]. CP can result in inadequate nutrition, poor quality housing, limited access to healthcare and trauma related to financial insecurity, all of which may impact physical and cognitive development [15]. As with ACEs, CP is linked to poorer health in later life including substance use, obesity, mental health issues, cardiovascular disease and other non-communicable diseases [16–19]. Even on a physiological level, both ACEs and CP have been associated with disruptions in the development of hormonal, immunological and neurological systems, leading to harmful chronic inflammation, increased allostatic load and consequent increased susceptibility to illness and premature death. The details of the physiological impacts of ACEs and CP are described in detail elsewhere [15, 20].

ACEs and CP are also consistently associated with poorer educational progression and subsequent economic and employment achievements. Thus, individuals with higher levels of ACEs are more likely to leave education with no qualifications, be in non- or low-paid employment and be resident in more deprived communities [21–23]. Similarly, individuals experiencing CP are more likely to have lower educational attainment and remain in poverty during adulthood [24, 25]. Determining how much life course health and economic hardship is related to ACEs and how much to CP is complicated by associations between the two. Children who live in poverty or reside in deprived communities are more likely to experience higher levels of ACEs [6, 26]. Moreover, the combined effects of experiencing ACEs and poverty may be more detrimental than experiencing either factor alone [5, 27].

Despite considerable work examining relationships between trauma in childhood and CP, relatively little empirical work has examined the full range of adversity measured by validated ACE tools and how together the burden of trauma from such ACEs and from CP interact to impact health, productivity and attainment across the life course. However, understanding, for instance, how CP contributes directly to life course health and attainment and how much is articulated through associated increases in ACEs, is an important consideration for developing health policies and practices and for understanding the economic repercussions of childhood experiences. Moreover, any adverse impacts of childhood experiences on life course health and economic attainment may also affect the socio-economic and family life experiences of subsequent generations. Thus, CP and ACEs may impact economic success, health and other factors relating to adversity in individual’s own children later in adult life. Understanding such intergenerational impacts is an important factor in breaking cycles of deprivation and adversity which can lock families into repeated patterns of adversity, ill health and underachievement. Here therefore, we use a large regional survey to measure the association between self-reported CP and ACE burden. To address the lack of studies examining how both ACEs and CP impact life course outcomes, we test the hypothesis that both are independently associated with poorer physical and mental health in adulthood. Distinctively, we also model the contributions that ACEs and CP have, independently and in association, to poorer economic and employment outcomes, and explore how interventions and policies may be developed to address the combined challenges posed by ACEs and CP.

## Methods

### Survey design

A cross-sectional household survey was conducted with adults (aged 18 years and over) in an English region (Merseyside) across five local authority/administrative areas, between November 2023 and April 2024. Stratified quota sampling was used to obtain a sample broadly matching the population for age, sex and deprivation. Lower Super Output Areas (LSOAs; small geography areas with a population of around 1,500) were used as the geographical sampling unit, stratified across local authorities by deprivation quintile. LSOAs were ranked by their score in the English Index of Multiple Deprivation (IMD [28]) and categorised into quintiles. The IMD is a standardised measure of deprivation. A total of 110 LSOAs were selected using a stratified random process to provide a sample matching the IMD for each local authority. A professional market research company (MRC) was procured to undertake recruitment and data collection. Quota samples by sex and age were set for each LSOA proportionate to area demographics. A target sample of 5,000 participants was set with study inclusion criteria being aged 18 years or over, resident in a household in a sampled LSOA, and cognitively able to participate.

Households within each selected LSOA were randomly selected and sent postal letters advertising the study. Letters provided study information, instructions on how to opt out and a link to take part in the survey online (using a unique reference number). Households that did not opt out or complete the survey online were then visited by trained interviewers from the MRC. On contact, residents were invited to complete the survey face-to-face using computer assisted personal interviewing technology, with computer assisted self-interviewing offered for sensitive questions. Household visits were made between 9am to 9 pm across all days of the week. Only one individual per household was eligible to participate. Where more than one resident was eligible, interviewers asked the person with the next birthday to take part. However, when individuals were no longer required to achieve age and sex quotas, alternatives were sampled according to quota need from the same household or a different household sampled as required. Where households were ineligible or declined to participate, the interviewer recorded the outcome of the contact and moved on to the next randomly selected household. Households were visited up to five times to elicit a response to participate or opt out. All participants were provided with a participant information sheet informing them of the voluntary, confidential and anonymous nature of the study, how their information would be used, and provided information on relevant support services. Informed consent was recorded from all participants as part of the survey.

In total, 54,761 postal letters were sent to selected households, of which 467 opted out and 1,215 completed the survey online. Subsequently, 6,040 households were contacted by a trained interviewer where an eligible participant answered the door, of whom 4,180 completed the survey. Thus, a total of 5,395 participants completed the survey.

### Measures and questionnaire design

All measures were self-reported and used existing, validated questions where possible. The survey measured participant demographics, CP, ACEs, and health and economic outcomes. Questions and response options used in this study are shown in Supplementary material Table A1. Supplementary material Table A1 also provides category boundary values for variables that are categorised for the purposes of analysis. The questionnaire was piloted by the MRC for length, flow and comprehension. The questionnaire took an average of 15 min to complete.

Exposure to nine types of ACE before the age of 18 (physical, verbal, or sexual abuse, parental separation/divorce, and household domestic violence, alcohol or drug misuse, mental illness, or incarceration) were measured using items from the Behavioral Risk Factor Surveillance System questionnaire [29] and the World Health Organization Adverse Childhood Experiences International Questionnaire [30]. Full details of the wording and scoring for these questions are provided in Supplementary material Table A1. These widely used measures focus on household adversity and have been shown to have acceptable psychometric properties [31, 32]. Despite the sensitive nature of ACE questions response rates to each ACE measure were high with ‘prefer not to say’ responses below 8.0% across all measures (physical 7.6%, verbal 7.3%, or sexual abuse 7.0%, parental separation/divorce 5.8%, and household domestic violence 7.7%, alcohol 5.2% or drug misuse 4.9%, mental illness 5.7%, or incarceration 4.8%).

Our main explanatory variables, as well as demographic measures, were CP and ACE count. CP was measured by asking participants how well off their household was during their childhood from 1 (very poor) to 10 (very wealthy) and therefore was a measure of how poor respondents perceived their living conditions during childhood. CP was then categorised into tertiles (poorest, mid, wealthiest; see Supplementary material Table A1). A categorical methodology was used for CP to allow independent relationships with ACE categories and with outcomes of interest to be measured for lower, mid, and highest self-reported poverty with no a priori assumptions about linear or ordinal relationships. This approach is discussed further in the study limitations. For our dependent health variables, we used two self-assessed measures of health. The first asked if respondents had a chronic condition, measured as reporting having a physical or mental health condition or illness lasting or expected to last for 12 months or more. The second, lower health was measured by asking participants how good or bad their health is today, from 0 (the worst health you can imagine) to 100 (the best health you can imagine) [33]. As the study aims to examine relationships with poorer physical and mental health, the health scale was categorised into tertiles (as with CP) and the bottom tertile considered lower health (see Supplementary material Table A1). The final dependent health variable measure examined mental well-being (MWB). This was measured using the validated Short Warwick-Edinburgh Mental Wellbeing Scale (SWEMWBS [34]). Adhering to SWEMWBS guidelines, scores across the seven items were summed and transformed to metric scores, and then categorised into tertiles consistent with the handling of other variables. Those in the bottom tertile were classified as having low MWB.

Our dependent economic outcome variables were reporting being unemployed or unable to work due to health reasons (unemployed/long-term sick), and low income (those reporting ≤ £20,000 yearly household income before deductions, based on the closest categorisation to national low income classifications [35]). As these measures of current economic status are outcome variables in this study they are not included as independent variables in statistical models. Demographic measures included participants’ sex, age (age bands categorised into age groups for analyses: 18–24, 25–44, 45–64 and 65 + years), and ethnicity (self-defined using UK census categories, see [36] for more on UK ethnicity categorisation). The MRC assigned all participants to an IMD deprivation quintile based on their LSOA of household residence.

### Methodological strategy and data analyses

Statistical analyses were undertaken in SPSS (v.28). Rather than use a continuous measure of ACE count or examining each individual type of ACE as a separate variable, we use a categorised measure of childhood adversity burden (ACE count categories, see Table 1). By adopting this approach, we aimed to calculate relationships between ACEs and outcomes largely consistent and comparable to other major ACE studies which use this methodology [7, 11, 37]. Thus, the total number of positive responses to ACE measures were summed to give an ACE count variable for analysis (0 ACEs, 1 ACE, 2–3 ACEs, and 4 + ACEs). Individual ACE positive response prevalences were physical 23.0%, verbal 23.5%, or sexual abuse 6.5%, parental separation/divorce 20.3%, and household domestic violence 15.7%, alcohol 13.1% or drug misuse 4.0%, mental illness 15.6% or incarceration 2.6%; see Supplementary material Table A1 for full ACE definitions). Due to small numbers reporting being other than white ethnicities, ethnicity was recategorized into white and other than white. For the purpose of analyses, we excluded respondents with missing demographic data (*n* = 65), leaving a final sample of 5,330 for analysis. Variations in sample due to individuals not completing each outcome variable are provided in tables.

**Table 1:** Relationships between self-reported childhood poverty, demographics and adverse childhood experiences

		**ACE count by category**				
		**0**	**1**	**2–3**	**4 + **	
**All**	n	2669	1018	1010	633	*X*^*2*^
	%	50.1	19.1	18.9	11.9	P
**Childhood poverty tertiles**	(poorest) 1	38.2	17.8	23.3	20.7	
	(mid) 2	55.1	19.2	17.7	8.0	279.337
	(wealthiest) 3	57.4	20.9	15.1	6.7	< 0.001
**Age (years)**	18–24	52.8	17.7	14.9	14.5	
	25–44	46.8	19.5	19.0	14.7	
	45–64	47.7	18.6	21.2	12.6	78.276
	65 +	56.4	19.7	17.4	6.4	< 0.001
**Sex**	Male	51.3	19.8	19.2	9.7	21.624
	Female	48.9	18.5	18.7	13.8	< 0.001
**Ethnicity**	White	49.2	19.5	19.2	12.0	21.690
	Other than white	61.4	13.3	15.2	10.1	< 0.001

*ACE* adverse childhood experience. For information on categorisation of variables see Supplementary material Table A1

**Fig. 1:**
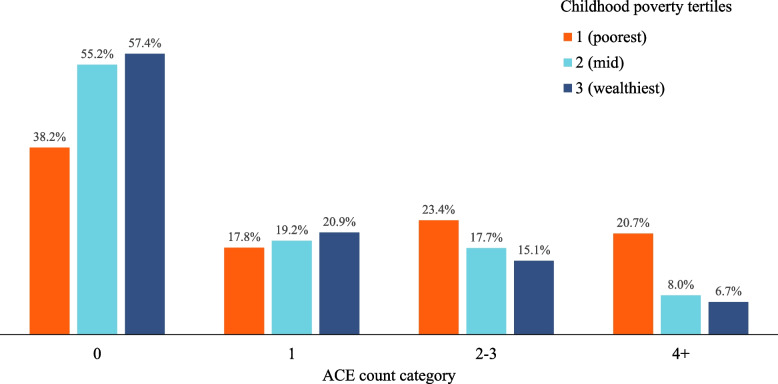
Adjusted percentage of individuals in each adverse childhood experience category by self-reported level of childhood poverty. Footnote: Figures are based on estimated marginal means generated using multinomial logistic models outlined in Table 2

Bivariate analyses used chi-squared tests to examine associations between demographics, CP and ACE count (Table 1) and multinomial logistic regression models (MLRM) were used to account for any potential demographic confounding of relationships between CP and ACE count (Table 2). Fitted MLRM were used to calculate adjusted proportions (estimated marginal means) within each ACE count category by CP tertile (Fig. 1). Similarly, chi-squared tests were used to examine relationships between health and economic outcomes (See Supplementary material Table A1) and CP, ACE counts and demographics (Table 3). Binomial logistic regression models (BLRM) were used to account for any demographic confounding and establish the independent contribution of predictive variables to the health and economic outcomes examined. Interaction terms between ACE count and CP were included in all models. However, as none were significant, they are included only in the footnote of Table 4. Fitted BLRM were used to calculate adjusted proportions (estimated marginal means) for each health and economic outcome by category of reported CP and ACE count (for Fig. 3).

**Table 2:** Multinomial logistic regression analysis of relationships between self-reported childhood poverty, demographics and adverse childhood experiences

		**ACE count**						
		**0 ACEs**	**1 ACE**		**2–3 ACEs**		**4 + ACEs**	
		Ref	AOR (95%CIs)	P	AOR (95%CIs)	P	AOR (95%CIs)	P
**Childhood poverty tertiles **	(poorest) 1		1.30 (1.07–1.59)	0.010	2.41 (1.96–2.97)	< 0.001	5.53 (4.23–7.23)	< 0.001
	(mid) 2		0.96 (0.80–1.15)	0.638	1.23 (1.01–1.50)	0.039	1.36 (1.03–1.80)	0.031
	(Ref; wealthiest) 3	P < 0.001						
**Age (years)**	18–24		1.07 (0.80–1.41)	0.658	1.10 (0.81–1.48)	0.550	3.32 (2.33–4.73)	< 0.001
	25–44		1.32 (1.09–1.60)	0.005	1.54 (1.26–1.89)	< 0.001	3.53 (2.69–4.65)	< 0.001
	45–64		1.16 (0.96–1.41)	0.122	1.55 (1.28–1.89)	< 0.001	2.66 (2.02–3.50)	< 0.001
	(Ref) 65 +	P < 0.001						
**Sex**	Male		1.03 (0.89–1.19)	0.735	0.97 (0.83–1.12)	0.648	0.66 (0.55–0.79)	< 0.001
	(Ref) Female	P < 0.001						
**Ethnicity**	White		1.96 (1.42–2.69)	0.001	1.67 (1.23–2.27)	0.001	1.85 (1.28–2.68)	0.001
	(Ref) Other than white	P < 0.001						

*ACE* adverse childhood experience, AOR = adjusted odds ratio, CIs = confidence intervals. Reference (Ref) category for ACEs is no ACEs. Other reference categories are identified next to each independent variable. For information on categorisation of variables seeSupplementary material Table A1

**Table 3:** Relationships between health and economic outcomes and ACE count, self-reported childhood poverty and current demographics

			**Health**			**Economic**	
			**Chronic health condition**	**Lower health tertile**	**Lower MWB tertile**	**Unemployed/long-term sick**	**Lower income**
**All**		n	5167	4973	5048	5330	5330
		%	30.1	36.6	38.3	10.5	20.0
**ACE count**		0	23.1	32.3	30.6	8.5	16.4
		1	29.5	36.2	36.4	9.4	20.8
		2–3	37.2	42.8	49.7	12.5	24.1
		4+	48.5	44.5	53.5	17.4	27.5
		*X*^*2*^	183.936	51.063	177.038	48.150	53.983
		P	< 0.001	< 0.001	< 0.001	< 0.001	< 0.001
**Childhood poverty tertiles**		(poorest) 1	38.3	47.7	46.2	14.6	26.1
		(mid) 2	26.8	33.3	36.5	9.1	17.9
		(wealthiest) 3	24.6	27.2	30.7	7.3	15.5
		*X*^*2*^	82.888	139.588	74.807	50.023	62.536
		P	< 0.001	< 0.001	< 0.001	< 0.001	< 0.001
**Age (years)**		18–24	15.2	20.5	41.0	11.0	18.5
		25–44	21.3	28.9	38.6	10.2	13.9
		45–64	34.2	41.3	38.3	17.7	20.0
		65 +	41.3	46.3	36.9	1.3	28.5
		*X*^*2*^	204.079	159.224	2.483	218.830	101.161
		P	< 0.001	< 0.001	0.478	< 0.001	< 0.001
**Sex**		Male	28.1	36.7	38.3	11.3	19.3
		Female	32.0	36.5	38.3	9.8	20.7
		*X*^*2*^	9.368	0.030	0.002	2.913	1.722
		P	0.002	0.862	0.962	0.088	0.189
**Ethnicity**		White	31.4	37.6	38.6	10.7	20.2
		Other than white	13.2	23.6	34.7	8.0	17.8
		*X*^*2*^	52.346	26.299	2.017	2.749	1.243
		P	< 0.001	< 0.001	0.156	0.097	0.265

See methods and Supplementary material Table A1 for definition and categorisation of each variable

**Table 4:** Adjusted odds ratios for health and economic outcomes by ACE count

		**Health**						**Economic**			
		**Chronic health condition**		**Lower health tertile**		**Lower MWB tertile**		**Unemployed/long-term sick**		**Lower income**	
		AOR (95%CIs)	P	AOR (95%CIs)	P	AOR (95%CIs)	P	AOR (95%CIs)	P	AOR (95%CIs)	P
**ACE count**	4 +	3.46 (2.84–4.22)	< 0.001	1.56 (1.28–1.90)	< 0.001	2.30 (1.91–2.77)	< 0.001	1.59 (1.22–2.07)	< 0.001	1.96 (1.58–2.43)	< 0.001
	2–3	2.00 (1.69–2.36)	< 0.001	1.46 (1.24–1.71)	< 0.001	2.11 (1.81–2.46)	< 0.001	1.25 (0.99–1.60)	0.065	1.60 (1.33–1.92)	< 0.001
	1	1.41 (1.19–1.67)	< 0.001	1.16 (0.99–1.36)	0.068	1.26 (1.08–1.48)	0.003	1.03 (0.80–1.33)	0.833	1.36 (1.13–1.64)	0.001
	(Ref) 0		< 0.001		< 0.001		< 0.001		0.003		< 0.001
**Childhood poverty tertiles**	(poorest) 1	1.37 (1.15–1.63)	< 0.001	2.03 (1.72–2.40)	< 0.001	1.64 (1.39–1.93)	< 0.001	2.19 (1.68–2.85)	< 0.001	1.58 (1.30–1.92)	< 0.001
	(mid) 2	0.97 (0.82–1.15)	0.718	1.21 (1.03–1.42)	0.020	1.27 (1.09–1.48)	0.002	1.28 (0.99–1.67)	0.062	1.09 (0.90–1.32)	0.356
	(Ref; wealthiest) 3		< 0.001		< 0.001		< 0.001		< 0.001		< 0.001
**Age (years)**	18–24	0.23 (0.18–0.31)	< 0.001	0.32 (0.24–0.41)	< 0.001	1.21 (0.97–1.51)	0.094	10.16 (5.87–17.56)	< 0.001	0.56 (0.43–0.72)	< 0.001
	25–44	0.34 (0.29–0.41)	< 0.001	0.49 (0.41–0.57)	< 0.001	1.04 (0.89–1.22)	0.610	9.08 (5.54–14.89)	< 0.001	0.38 (0.31–0.45)	< 0.001
	45–64	0.67 (0.57–0.78)	< 0.001	0.82 (0.70–0.95)	0.008	1.01 (0.86–1.18)	0.924	16.60 (10.24–26.91)	< 0.001	0.59 (0.50–0.70)	< 0.001
	(Ref) 65 +		< 0.001		< 0.001		0.358		< 0.001		< 0.001
**Sex**	Male	0.80 (0.70–0.91)	< 0.001	0.97 (0.86–1.09)	0.560	1.01 (0.90–1.14)	0.852	1.24 (1.03–1.49)	0.021	0.87 (0.76–1.00)	0.055
**Ethnicity**	Other than white	2.18 (1.58–3.03)	< 0.001	1.48 (1.13–1.94)	0.004	1.12 (0.88–1.42)	0.348	1.50 (1.01–2.22)	0.043	0.86 (0.65–1.14)	0.297

*ACE* adverse childhood experience, *MWB* mental wellbeing, *AOR* adjusted odds ratio, *CIs* confidence intervals, *Ref* reference category. *P* values refer to difference between Ref category and other categories for each variable. P values for Ref categories identify level of significance variable contributes to model. Ref categories for Sex and Ethnicity are female and other than white respectively. Interactions between ACE count and childhood poverty tertiles were included in the models but none reached significance (Chronic health condition, P = 0.963; Lower health tertile, 0.084; Lower MWB tertile, 0.679; Unemployed or long-term sick, 0.897; Lower income, 0.628) and therefore interactive terms have not been presented

**Fig. 2:**
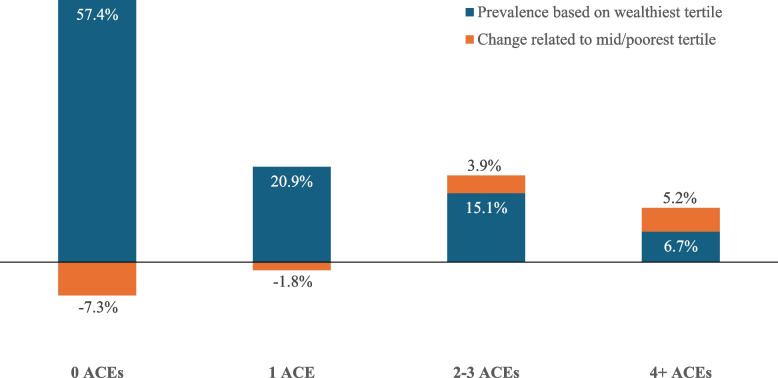
Contribution of increases in childhood poverty to membership of different ACE count categories. Footnote: Estimated changes in proportions of individuals with different ACE counts were calculated by applying ACE proportions from the wealthiest tertile to all tertiles and subtracting wealthiest modelled levels from overall modelled estimates. Positive changes in ACE count category indicate the increased percentage in that ACE category resulting from mid/poorest CP tertile membership. Negative changes indicate how much the total percentage is reduced by mid/poorest CP tertile membership

The proportion of ACEs associated specifically with increased CP was also calculated using the proportion of individuals with each ACE count within each CP tertile (Fig. 1). Estimated changes in proportions of individuals with different levels of ACE counts (for Fig. 2) were calculated by applying ACE proportions from the wealthiest tertile to all tertiles and subtracting levels modelled using wealthiest tertile levels from overall modelled estimates.

### Ethical approval

Ethical approval was granted for the study by Liverpool John Moores Research Ethics Committee (23/PHI/050).

## Results

Sample demographics are provided in Supplementary material Table A2 and the proportion of participants within each ACE count category is shown in Table 1.

### Relationships between ACE count and childhood poverty

ACE count was significantly related to CP tertile in both bivariate and MLRM, with adjusted odds of having 4 + ACEs being over five times higher in those from the poorest CP tertile compared with the wealthiest (Tables 1 and 2). Age, sex and ethnicity were also significantly associated with ACE count, with reporting 4 + ACEs most likely in those aged 18–44 years, females and those of white ethnicity (Tables 1 and 2). Using the MLRM identified in Table 2, adjusted means were calculated for different percentages of ACEs by CP tertile (Fig. 1). Percentages with 0 ACEs or 1 ACE decreased with increasing CP category whilst percentages with 2–3 or 4 + ACEs increased. Thus, 4 + ACEs rose from 6.7% from the wealthiest to 20.7% from the poorest CP tertile (Fig. 1). Estimated increases in ACE counts relating to CP (see Methods) were calculated by subtraction of modelled counts based on the whole sample experiencing the wealthiest CP tertile from the fully CP adjusted totals (in Fig. 1). Figure 2 shows that both 2–3 and 4 + ACE categories increase substantively with increased CP. Thus, of the overall 11.9% of individuals reporting 4 + ACEs, 5.2% is associated with the increase in ACEs associated with mid or poorest CP tertile membership. Expressed as a percentage of only those with 4 + ACEs, 43.7% of 4 + ACEs reports are accounted for by changes in CP category. By contrast, increases in CP are associated with decreases in the 0 and 1 ACE category (Fig. 2).

### ACEs, childhood poverty, and health

**Fig. 3:**
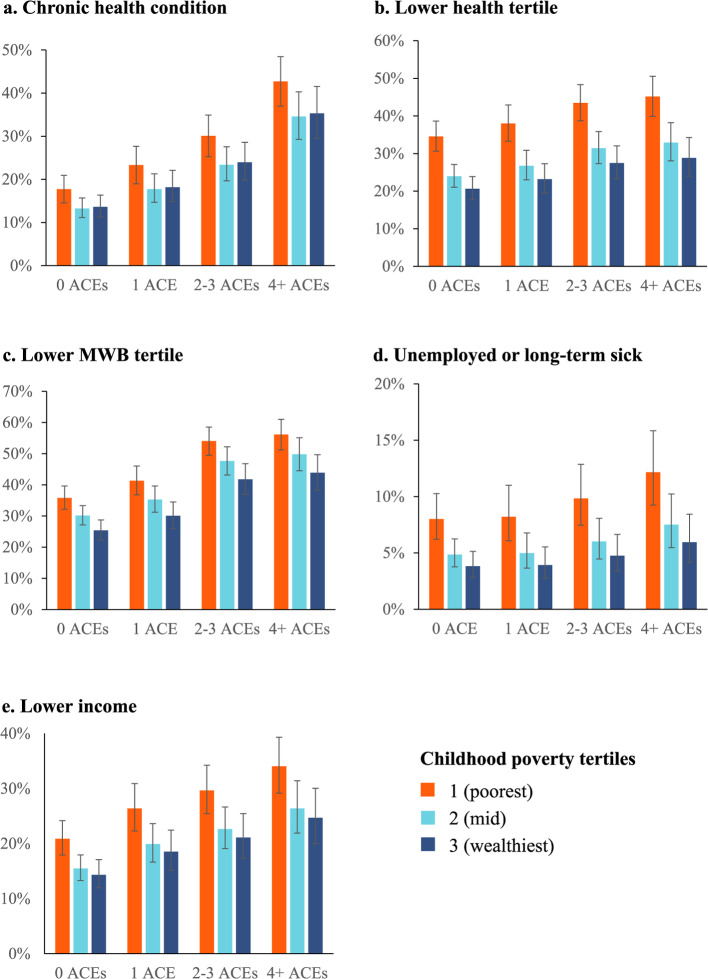
Adjusted relationships between ACE count, childhood poverty and health and economic outcomes. Footnote: Figures are based on estimated marginal means generated using binary logistic models outlined in Table 4

Bivariate and multivariate analyses were undertaken to examine relationships between number of ACEs, CP and adult health (i.e. chronic health condition, lower health tertile and lower MWB tertile). ACE count and CP both showed significant ordinal relationships with all three health outcomes. Thus, increasing ACE count category and poorer CP tertile were both related to higher proportions of individuals reporting a chronic health condition, and lower health tertile and lower MWB tertile membership (Table 3). These relationships were still significant when both variables and socio-economic factors were included in BLRM (Table 4). Thus, likelihood of being in the lower health tertile was 1.56 times higher in those with 4 + ACEs (vs. 0 ACEs) with, independently, 2.03 times higher likelihood in those reporting being in the poorest CP tertile (vs. wealthiest; Table 4). Adjusted means (see Methods) showed a rise in percentage in lower health tertile membership from 20.6% in those with 0 ACEs and from the wealthiest CP tertile to 45.2% in those with 4 + ACEs and from the poorest CP tertile (Fig. 3b). People from the poorest CP tertile with 0 ACEs or 4 + ACEs and from the wealthiest CP tertile showed intermediate values (34.6% and 28.9% respectively). Chronic health condition and lower MWB tertile membership equivalent figures showed similar patterns with highest percentages for both outcomes with 4 + ACEs from the poorest CP tertile and intermediate values when only one such risk factor was reported (Fig. 3a, c). Across health outcomes, all categories of ACE count and CP were significantly different from their reference categories (0 ACEs, wealthiest CP tertile respectively) except for the mid CP tertile for chronic health condition and the 1 ACE category for lower health tertile membership (Table 4). Prevalence of having a chronic health condition and being in the lower health tertile increased significantly with age. However, being in the lower MWB tertile was not significantly associated with age. Only prevalence of a chronic health condition was significantly associated with sex (higher in females). People of white ethnicity were more likely to report having a chronic health condition or lower health tertile membership (Tables 3 and 4). Sensitivity analyses were run for each health outcome limiting the data to those aged less than 65 years (Supplementary material Table A3). Findings from these show independent relationships between health outcomes and both CP and ACEs that are consistent with the wider sample.

### ACEs, childhood poverty, and economic outcomes

Both of the adult economic outcomes examined (unemployed/long-term sick, lower income) were strongly associated with being from the poorest CP tertile and higher ACE count categories (Table 3). Using BLRM, unemployed/long-term sick and lower income remained significantly associated with higher ACE count categories and the poorest CP tertile (Table 4). However, whilst 4 + ACE count categories for both variables were significantly different (vs. 0 ACEs), 1 and 2–3 ACE count categories failed to reach significance for unemployed/long-term sick. Equally the mid CP tertile was not significantly different from the wealthiest for both outcomes (Table 4). Adjusted means show a rise in percentage unemployed/long-term sick from 3.8% in those from the wealthiest CP tertile and experiencing 0 ACEs to 12.2% in those from the poorest CP tertile with 4 + ACEs (Fig. 3d) with equivalent figures for lower income being 14.3% and 34.0% respectively (Fig. 3e). Both economic outcomes were strongly related to age with unemployed/long-term sick highest in those age 45–64 years and lower income most likely in those aged 65 years or older (Tables 3 and 4). Males and those of other than white ethnicity were also more likely to report being unemployed/long-term sick but neither variable was significantly related to lower income. As with health outcomes, sensitivity analyses (sample limited to < 65 years) were also run for each economic outcome which identified relationships with CP and ACEs consistent with the whole sample (Supplementary material Table A3).

## Discussion

Findings here suggest ACEs are common across all socio-economic backgrounds but significantly more frequent in those with greater CP. Both higher numbers of ACEs and greater self-assessed CP were independently associated with a greater likelihood of worse health and economic outcomes in adulthood, resulting in those exposed to both CP and ACEs being at greatest risks of poor life course outcomes. These findings and their potential policy implications are explored further in the following sections along with the study limitations.

### Associations between ACEs and CP

Our findings are consistent with other studies examining associations between CP and ACEs [6, 26] and demonstrate strong relationships between self-reported greater CP and increased risks of experiencing greater numbers of ACEs during childhood. Membership of the poorest and mid poverty CP tertiles accounted for a 43.7% increase in the 4 + ACEs and 20.5% increase in the 2–3 ACEs categories compared to levels of ACE exposure based on all individuals having experienced the wealthiest CP tertile (Fig. 2). Parents with less resources are more likely to be in poor health than wealthier counterparts [38] and may have less opportunities and ability to implement more beneficial parenting options [39]. Consequently, they may expose children to higher levels of ACEs. More beneficial parenting options may be even more difficult to implement when children have physical or mental health conditions which place additional pressures on parents. Equally, our CP measure was based on a single question measuring self-perceived CP and we cannot identify here specifically whether its associations with ACEs are directly due to lower income, or through related issues such as the impacts of income on access to services. However, social protection policies to prevent poverty and provide more equitable distribution of resources (e.g. social insurance and cash transfer schemes) are likely to reduce both CP and the associated risks of ACEs [40, 41].

### Associations of CP and ACEs with life course outcomes

Results here identify an additive negative impact of ACEs and CP on life course outcomes. Thus, both factors appear to contribute independently to poorer health outcomes. Further, the absence of any significant interaction between ACEs and CP in LR models (see Table 4 footnote) suggests ACEs are associated with similar increases in likelihood of poorer health outcomes at each level of CP. Therefore, those experiencing ACEs at any level of CP experience are more likely to report poorer health outcomes than those with no ACEs, with the highest likelihood of such outcomes reported by those in the poorest tertile of CP with high ACE counts (Fig. 3a-c). Here for instance, lower MWB tertile membership increased with ACEs from 25.4% (0 ACEs) to 43.9% (4 + ACEs) in those from the wealthiest CP tertile but from a higher baseline of 35.8% to 56.2% in those from the poorest tertile (Fig. 3c). Equivalent figures for lower adult health tertile membership were 20.6% (0 ACEs) to 28.9% (4 + ACEs) in those from the wealthiest CP tertile and 34.6% to 45.2% in those from the poorest CP tertile (Fig. 3b).

As well as health measures this study examined outcomes related to employment and income. As with health outcomes, results suggest significant independent and consequently additive associations of both CP and ACEs with both economic measures (Fig. 3d, e). Such independent relationships were maintained when analyses were limited to those under 65 years of age (Supplementary material Table A3). Across the whole sample, being unemployed/long-term sick increased from 3.8% (0 ACEs) to 5.9% (4 + ACEs) in those from the wealthiest CP tertile but from 8.0% to 12.2% in those from the poorest (Fig. 3d). Equivalent figures for low income were 14.3% to 24.7% (0 ACEs) and 20.9% to 34.0% (4 + ACEs) respectively (Fig. 3e). Findings are consistent with CP and ACEs contributing to an intergenerational cycle of poverty and childhood adversity. Thus, whilst CP is associated with higher levels of ACEs, those with more ACEs were also more likely to report lower incomes and employment in adulthood and consequently are likely to contribute to CP and increased risk of ACEs in subsequent generations. Importantly, even 1 ACE (vs. 0 ACEs) resulted in a significant increase in reporting lower adult income (Table 4).

### Policy implications

Studies of this nature cannot establish causality. Poorer physical and mental health in childhood may result from ACEs but may also result from other genetic and environmental causes, with ACEs subsequently occurring potentially as a result of the additional pressures which having ill children places on parents. However, results here are only part of a growing body of evidence supporting causal relationships between exposure to ACEs and CP and poorer health and other outcomes in childhood and across the life course [42, 43]. Elsewhere, for ACEs a great deal of attention has been paid to those with the highest ACE counts (typically 4 + ACEs). Here however likelihood of lower MWB and reporting having a chronic health condition increased substantively even in those with 1 ACE (Table 4). Given that half of all people sampled here reported at least 1 ACE, a figure consistent with other surveys in the UK and abroad [8, 37], ACEs and their impacts should be considered a public health crisis.

Findings suggest that structural interventions that help reduce levels of CP may also help to reduce ACEs. However, results also indicate that such interventions would not eliminate the majority of ACEs. Interventions that provide support to parents and children during early years have been shown to reduce risk of, and impacts from ACEs, helping build both child rearing skills in parents and resilience to the impacts of ACEs in children [44, 45]. Results here are consistent with adopting a proportionate universalism model for the deployment of such assets and services recognising the additional risks and requirement of those born into CP but acknowledging that ACEs are experienced across socio-economic scales. Identification and early intervention with children at risk of ACEs in all communities is another critically important element in providing support and altering the life course trajectory of their health. Such identification and intervention require system wide trauma informed approaches in health, educational and judicial services so that all opportunities to identify those at risk and provide support are seized and systems work together to coordinate interventions [46, 47].

The economic benefits of investing in safe, supported and secure childhoods could be profound and propagate across generations. Estimates of the cost relating to health and well-being impacts of ACEs are $581 billion annually for Europe [48] and more recent estimates for the wider costs to the USA suggest an annual cost of $14.1 trillion [49]. Equally, estimates suggest the annual cost of CP in the USA is over $1 trillion, including costs related to lost economic productivity, health, crime, child homelessness and maltreatment [50]. A fraction of these costs invested in the prevention of ACEs, the development of resilience and addressing other aspects of CP are likely to provide substantive returns on investment, both in the shorter-term through impacts on schools, juvenile anti-social behaviour and lower immediate child ill health costs, as well as longer-term returns through lower chronic ill health and better MWB. In addition, returns should continue as individuals secure better employment and income; contributing to national revenues and reducing risks of CP and ACE related costs in subsequent generations.

### Limitations and further research

Data collected here were retrospective and included self-assessed measures of ACEs, CP and both health and economic outcomes. Simple measures of self-assessed health and self-reported measures of functional ability and disability have been found to be stronger predictors of, for instance, survival than biomarkers [51] and wherever possible we employed validated, routinely used questions. However, we cannot rule out impacts on findings from participants not remembering or identifying items correctly or not wishing to report childhood experiences. Our use of retrospective data collection on childhood has been widely employed in large studies and our results (e.g. ACE counts) are similar to those reported elsewhere [8, 37]. Whilst participation of households at the face-to-face visit level was relatively high (69.2%) compared to similar studies [8], we cannot rule out any selection bias introduced into the data by those who declined to participate. The offer of computer assisted self-interviewing for sensitive questions may have also contributed to high response rates for ACE questions.

Longitudinal studies are needed to further explore relationships between CP, ACEs and life course outcomes, but will continue to face ethical considerations including identification of children still being abused and their continued participation in studies. We only used one measure of CP and this was self-assessed and consequently a perceived measure of CP. In contrast, other studies have employed survey data and administrative data sources to examine family poverty during childhood and its relationships with intergenerational poverty [52]. Such studies often utilise monetary indicators such as family income collected through surveys or government recorded income data. These can provide objective financial metrics which in some cases can be tracked over multiple years. However, more subjective measures can also help capture non-financial factors such as parental behaviours, preferences and styles which may modify the impacts of income on experienced CP [52, 53]. More detailed measures of CP should be employed in future studies of ACEs and CP which consider both financial measures and perceived, self-assessed measures of CP. Our perceived CP measure was categorised into tertiles. Categorical methods were preferred in these analyses as neither linear nor other ordinal relationships were assumed between dependent and independent variables. However, we cannot exclude within category variations in relationships between CP, ACEs and the outcomes examined in this study. More detailed measures of CP should be employed in future studies. Whilst the current study had a relatively large sample size, it was only designed to be broadly representative of a single English region. Thus, although the sample included individuals across age, sex, economic and ethnic groups, the applicability of findings to other geographies require testing.

Further research is needed to better understand how health and socio-economic outcomes are impacted by CP and individual ACEs. For this initial study we adopted a cumulative ACE count (see Methods) which provides comparable results with other large ACE studies. However, individual ACEs such as physical abuse and exposure to domestic violence may interact differently with CP, resulting in a range of impacts on life course outcomes. Despite a relatively large sample size, we were not able here to identify if or how individual or combined impacts of CP and ACEs may vary with cohort, ethnicity and poverty. Larger studies and combinations of studies similar to the one conducted here should be used to examine such issues. In our study respondents reported CP and ACEs experienced across all years under the age of 18. Whilst this is consistent with other ACE studies, exposure to ACEs and experience of CP may both have varied across this age range, potentially with differing effects on health and economic outcomes across the life course. Equally, larger or multiple studies should examine the impact on findings of using smaller category ranges where there may also be differences in health and economic status within, for instance, each age band.

## Conclusions

This study suggests strong associations between CP and increased risks of growing up with higher numbers of ACEs. Despite the occurrence of CP and ACEs being strongly linked, results also support them contributing independently to health outcomes across the life course. Thus, those from poorer childhoods and also with high levels of ACEs had the highest risks of a chronic health condition and lower MWB. With an established evidence base of policies and interventions available to address CP and ACEs, the life-long harms associated with them are avoidable and investing in better early years is likely to provide positive returns on investments. ACEs and CP also appear to contribute to lower income and less stable employment in adulthood, meaning early years investments may also help break intergenerational cycles of adversity and poverty. Services that understand trauma are also an important feature of supporting with those with traumatic childhoods. Together, findings underscore the need for multi-sectoral trauma informed approaches that integrate health, education, criminal justice and social sectors operating within an equity-based framework. Although this was a UK regional study, the nature of these challenges are reflected internationally and results here should be considered part of a growing body of evidence highlighting how investing too little in our children can result in sicklier, less productive and poorer adults.

## Supplementary Information

Supplementary Material 1

## Data Availability

The dataset analysed in the current study is available from the corresponding author on reasonable request.

## Abbreviations

ACE: Adverse childhood experience
AOR: Adjusted odds ratio
BLRM: Binomial logistic regression models
CI: Confidence interval
CP: Childhood poverty
IMD: Index of Multiple Deprivation
LSOA: Lower Super Output Area
MLRM: Multinomial logistic regression models
MRC: Market Research Company
MWB: Mental well-being
Ref: Reference category
SWEMWBS: Short Warwick-Edinburgh Mental Wellbeing Scale

## References

[1] Karatekin C, Mason SM, Riegelman A, Bakker C, Hunt S, Gresham B, et al. Adverse childhood experiences: a scoping review of measures and methods. Child Youth Serv Rev. 2022;136: 106425. 10.1016/j.childyouth.2022.106425.

[2] Warner TD, Leban L, Pester DA, Walker JT. Contextualizing adverse childhood experiences: the intersections of individual and community adversity. J Youth Adolesc. 2023;52:570–84. 10.1007/s10964-022-01713-2. 36445650 10.1007/s10964-022-01713-2

[3] Hughes K, Bellis MA, Ford K, A Sharp C, Hopkins J, Hill R, et al. Adverse childhood and school experiences: a retrospective cross-sectional study examining their associations with health-related behaviours and mental health. BMC Public Health. 2025;25:672. 10.1186/s12889-025-21788-3. 10.1186/s12889-025-21788-3 PMC11837321 39966866

[4] Bernard DL, Smith Q, Lanier P. Racial discrimination and other adverse childhood experiences as risk factors for internalizing mental health concerns among Black youth. J Trauma Stress. 2022;35:473–83. 10.1002/jts.22760. 34800051 10.1002/jts.22760 PMC9035019

[5] Lee H, Slack KS, Berger LM, Mather RS, Murray RK. Childhood poverty, adverse childhood experiences, and adult health outcomes. Health Soc Work. 2021. 10.1093/hsw/hlab018. 34312679 10.1093/hsw/hlab018

[6] Walsh D, McCartney G, Smith M, Armour G. Relationship between childhood socioeconomic position and adverse childhood experiences (ACEs): a systematic review. J Epidemiol Community Health. 2019;73:1087–93. 10.1136/jech-2019-212738. 31563897 10.1136/jech-2019-212738 PMC6872440

[7] Merrick MT, Ford DC, Ports KA, Guinn AS, Chen J, Klevens J, et al. Vital signs: estimated proportion of adult health problems attributable to adverse childhood experiences and implications for prevention — 25 states, 2015–2017. MMWR Morb Mortal Wkly Rep. 2019;68:999–1005. 10.15585/mmwr.mm6844e1. 31697656 10.15585/mmwr.mm6844e1 PMC6837472

[8] Hughes K, Ford K, Kadel R, Sharp CA, Bellis MA. Health and financial burden of adverse childhood experiences in England and Wales: a combined primary data study of five surveys. BMJ Open. 2020;10: e036374. 10.1136/bmjopen-2019-036374. 32513892 10.1136/bmjopen-2019-036374 PMC7282338

[9] Graf GH-J, Chihuri S, Blow M, Li G. Adverse childhood experiences and justice system contact: a systematic review. Pediatrics. 2021;147:2020021030. 10.1542/peds.2020-021030. 10.1542/peds.2020-021030 PMC7786827 33328338

[10] Taillieu TL, Davila IG, Struck S. ACEs and violence in adulthood. In: Asmundson GJG, Afifi TO, editors. Adverse childhood experiences: using evidence to advance research, practice, policy, and prevention. Elsevier; 2020. p. 119–42. 10.1016/B978-0-12-816065-7.00007-0.

[11] Hughes K, Bellis MA, Hardcastle KA, Sethi D, Butchart A, Mikton C, et al. The effect of multiple adverse childhood experiences on health: a systematic review and meta-analysis. Lancet Public Health. 2017;2:e356–66. 10.1016/S2468-2667(17)30118-4. 29253477 10.1016/S2468-2667(17)30118-4

[12] Merrick MT, Ports KA, Ford DC, Afifi TO, Gershoff ET, Grogan-Kaylor A. Unpacking the impact of adverse childhood experiences on adult mental health. Child Abuse Negl. 2017;69:10–9. 10.1016/j.chiabu.2017.03.016. 28419887 10.1016/j.chiabu.2017.03.016 PMC6007802

[13] Alley J, Gassen J, Slavich GM. The effects of childhood adversity on twenty-five disease biomarkers and twenty health conditions in adulthood: differences by sex and stressor type. Brain Behav Immun. 2025;123:164–76. 10.1016/j.bbi.2024.07.019. 39025418 10.1016/j.bbi.2024.07.019 PMC11624074

[14] Melchior M, Moffitt TE, Milne BJ, Poulton R, Caspi A. Why do children from socioeconomically disadvantaged families suffer from poor health when they reach adulthood? A life-course study. Am J Epidemiol. 2007;166:966–74. 10.1093/aje/kwm155. 17641151 10.1093/aje/kwm155 PMC2491970

[15] Schmidt KL, Merrill SM, Gill R, Miller GE, Gadermann AM, Kobor MS. Society to cell: how child poverty gets “under the skin” to influence child development and lifelong health. Dev Rev. 2021;61: 100983. 10.1016/j.dr.2021.100983.

[16] Manhica H, Straatmann VS, Lundin A, Agardh E, Danielsson A. Association between poverty exposure during childhood and adolescence, and drug use disorders and drug-related crimes later in life. Addiction. 2021;116:1747–56. 10.1111/add.15336. 33197093 10.1111/add.15336 PMC8247994

[17] Galobardes B, Smith GD, Lynch JW. Systematic review of the influence of childhood socioeconomic circumstances on risk for cardiovascular disease in adulthood. Ann Epidemiol. 2006;16:91–104. 10.1016/j.annepidem.2005.06.053. 16257232 10.1016/j.annepidem.2005.06.053

[18] González D, Nazmi A, Victora CG. Childhood poverty and abdominal obesity in adulthood: a systematic review. Cad Saude Publica. 2009;25(suppl 3):S427–40. 10.1590/s0102-311x2009001500008. 20027390 10.1590/s0102-311x2009001500008

[19] Lee H, Slack KS, Berger LM, Mather RS, Murray RK. Childhood poverty, adverse childhood experiences, and adult health outcomes. Health Soc Work. 2021;46:159–70. 10.1093/hsw/hlab018. 34312679 10.1093/hsw/hlab018

[20] Berens AE, Jensen SKG, Nelson CA. Biological embedding of childhood adversity: from physiological mechanisms to clinical implications. BMC Med. 2017;15:135. 10.1186/s12916-017-0895-4. 28724431 10.1186/s12916-017-0895-4 PMC5518144

[21] Hardcastle K, Bellis MA, Ford K, Hughes K, Garner J, Ramos RG. Measuring the relationships between adverse childhood experiences and educational and employment success in England and Wales: findings from a retrospective study. Public Health. 2018;165:106–16. 10.1016/j.puhe.2018.09.014. 30388488 10.1016/j.puhe.2018.09.014

[22] Metzler M, Merrick MT, Klevens J, Ports KA, Ford DC. Adverse childhood experiences and life opportunities: shifting the narrative. Child Youth Serv Rev. 2017;72:141–9. 10.1016/j.childyouth.2016.10.021. 37961044 10.1016/j.childyouth.2016.10.021 PMC10642285

[23] Hughes K, Bellis MA, Cresswell K, Hill R, Ford K, Hopkins JC. Examining relationships between adverse childhood experiences and coping during the cost-of-living crisis using a national cross-sectional survey in Wales. UK BMJ Open. 2024;14: e081924. 10.1136/bmjopen-2023-081924. 38692715 10.1136/bmjopen-2023-081924 PMC11086514

[24] Liu J, Peng P, Zhao B, Luo L. Socioeconomic status and academic achievement in primary and secondary education: a meta-analytic review. Educ Psychol Rev. 2022;34:2867–96. 10.1007/s10648-022-09689-y.

[25] Parolin Z, Pintro-Schmitt R, Esping-Andersen G, Fallesen P. Intergenerational persistence of poverty in five high-income countries. Nat Hum Behav. 2024;9:254–67. 10.1038/s41562-024-02029-w. 39468279 10.1038/s41562-024-02029-w PMC11860229

[26] Farooq B, Allen K, Russell AE, Howe LD, Mars B. The association between poverty and longitudinal patterns of adverse childhood experiences across childhood and adolescence: findings from a prospective population-based cohort study in the UK. Child Abuse Negl. 2024;156: 107014. 10.1016/j.chiabu.2024.107014. 39232377 10.1016/j.chiabu.2024.107014

[27] Crouch E, Radcliff E, Hung P, Bennett K. Challenges to school success and the role of adverse childhood experiences. Acad Pediatr. 2019;19:899–907. 10.1016/j.acap.2019.08.006. 31401231 10.1016/j.acap.2019.08.006

[28] Ministry of Housing, Communities & Local Government. English indices of deprivation 2019. https://www.gov.uk/government/statistics/english-indices-of-deprivation-2019. Accessed 28 March 2025.

[29] Centers for Disease Control and Prevention. Behavioral Risk Factor Surveillance System ACE data. https://www.cdc.gov/violenceprevention/aces/ace-brfss.html. Accessed 28 March 2025.

[30] World Health Organization. Adverse childhood experiences international questionnaire (ACE-IQ). https://www.who.int/publications/m/item/adverse-childhood-experiences-international-questionnaire-(ace-iq). Accessed 28 March 2025.

[31] Ford DC, Merrick MT, Parks SE, Breiding MJ, Gilbert LK, Edwards VJ, et al. Examination of the factorial structure of adverse childhood experiences and recommendations for three subscale scores. Psychol Violence. 2014;4(4):432–44. 10.1037/a0037723. 26430532 10.1037/a0037723 PMC4587306

[32] Mei X, Li J, Li Z-S, Huang S, Li L-L, Huang Y-H, et al. Psychometric evaluation of an adverse childhood experiences (ACEs) measurement tool: an equitable assessment or reinforcing biases? Health Justice. 2022;10:34. 10.1186/s40352-022-00198-2. 36445502 10.1186/s40352-022-00198-2 PMC9706892

[33] EuroQol Group. EuroQol - a new facility for the measurement of health-related quality of life. Health Policy. 1990;16:199–208. 10.1016/0168-8510(90)90421-9. 10109801 10.1016/0168-8510(90)90421-9

[34] NHS Health Scotland, University of Warwick, University of Edinburgh. Short Warwick Edinburgh Mental Wellbeing Scale (SWEMWBS). 2008. https://www.corc.uk.net/outcome-experience-measures/short-warwick-edinburgh-mental-wellbeing-scale-swemwbs/. Accessed 28 March 2025.

[35] Office for National Statistics. Average household income, UK: financial year ending 2024. 2025. https://www.ons.gov.uk/peoplepopulationandcommunity/personalandhouseholdfinances/incomeandwealth/bulletins/householddisposableincomeandinequality/financialyearending2024. Accessed 6 June 2025.

[36] Office for National Statistics. Ethnic group classifications: Census 2021. 2023. https://www.ons.gov.uk/census/census2021dictionary/variablesbytopic/ethnicgroupnationalidentitylanguageandreligionvariablescensus2021/ethnicgroup/classifications. Accessed 6 June 2025.

[37] Madigan S, Deneault A-A, Racine N, Park J, Thiemann R, Zhu J, et al. Adverse childhood experiences: a meta-analysis of prevalence and moderators among half a million adults in 206 studies. World Psychiatry. 2023;22:463–71. 10.1002/wps.21122. 37713544 10.1002/wps.21122 PMC10503911

[38] Reed HR, Nettle D, Parra-Mujica F, Stark G, Wilkinson R, Johnson MT, et al. Examining the relationship between income and both mental and physical health among adults in the UK: analysis of 12 waves (2009–2022) of Understanding Society. PLoS One. 2025;20: e0316792. 10.1371/journal.pone.0316792. 40048442 10.1371/journal.pone.0316792 PMC11884696

[39] World Health Organization. Preventing violence against children: a social determinants framework for INSPIRE implementation. 2025. https://iris.who.int/bitstream/handle/10665/381460/9789240103191-eng.pdf. Accessed 8 June 2025.

[40] Puls HT, Hall M, Anderst JD, Gurley T, Perrin J, Chung PJ. State spending on public benefit programs and child maltreatment. Pediatrics. 2021;148: e2021050685. 10.1542/peds.2021-050685. 34663680 10.1542/peds.2021-050685

[41] Maguire-Jack K, Johnson-Motoyama M, Parmenter S. A scoping review of economic supports for working parents: the relationship of TANF, child care subsidy, SNAP, and EITC to child maltreatment. Aggress Violent Behav. 2022;65: 101639. 10.1016/j.avb.2021.101639.

[42] Bhushan D, Kotz K, McCall J, Wirtz S, Gilgoff R, Dube SR, et al. Roadmap for resilience: The California Surgeon General’s report on adverse childhood experiences, toxic stress, and health. Office of the California Surgeon General. 2020. https://osg.ca.gov/wp-content/uploads/sites/266/2020/12/Roadmap-For-Resilience_CA-Surgeon-Generals-Report-on-ACEs-Toxic-Stress-and-Health_12092020.pdf Accessed 8 June 2025.

[43] Le Menestrel S, Duncan G, eds. A roadmap to reducing child poverty. 3 – Consequences of child poverty. Washington (DC): National Academies Press; 2019. https://www.ncbi.nlm.nih.gov/books/NBK547371/. Accessed 8 June 2025.

[44] Maguire-Jack K, Hardi F, Stormer B, Lee JY, Feely M, Rostad W, et al. Early childhood education and care policies in the U.S. and their impact on family violence. Child Youth Serv Rev. 2022;142: 106653. 10.1016/j.childyouth.2022.106653. 10.1016/j.childyouth.2022.106653 PMC11177322 38881540

[45] Chen M, Chan KL. Effects of parenting programs on child maltreatment prevention. Trauma Violence Abuse. 2016;17:88–104. 10.1177/1524838014566718. 25573846 10.1177/1524838014566718

[46] ACE Hub Wales. Trauma-informed Wales: a societal approach to understanding, preventing and supporting the impacts of trauma and adversity. Cardiff; 2022. https://traumaframeworkcymru.com/. Accessed 28 March 2025.

[47] Tebes JK, Champine RB, Matlin SL, Strambler MJ. Population health and trauma-informed practice: implications for programs, systems, and policies. Am J Community Psychol. 2019;64:494–508. 10.1002/ajcp.12382. 31444915 10.1002/ajcp.12382 PMC7006880

[48] Bellis MA, Hughes K, Ford K, Ramos Rodriguez G, Sethi D, Passmore J. Life course health consequences and associated annual costs of adverse childhood experiences across Europe and North America: a systematic review and meta-analysis. Lancet Public Health. 2019;4:e517–28. 10.1016/S2468-2667(19)30145-8. 31492648 10.1016/S2468-2667(19)30145-8 PMC7098477

[49] Peterson C, Aslam MV, Niolon PH, Bacon S, Bellis MA, Mercy JA, et al. Economic burden of health conditions associated with adverse childhood experiences among US adults. JAMA Netw Open. 2023;6: e2346323. 10.1001/jamanetworkopen.2023.46323. 38055277 10.1001/jamanetworkopen.2023.46323 PMC10701608

[50] McLaughlin M, Rank MR. Estimating the economic cost of childhood poverty in the United States. Soc Work Res. 2018;42:73–83. 10.1093/swr/svy007.

[51] Goldman N, Glei DA, Weinstein M. The best predictors of survival: do they vary by age, sex, and race? Popul Dev Rev. 2017;43:541–60. 10.1111/padr.12079. 29398738 10.1111/padr.12079 PMC5791760

[52] Parolin Z, Pintro-Schmitt R, Esping-Andersen G, Fallesen P. Intergenerational persistence of poverty in five high-income countries. Nat Hum Behav. 2025;9:254–67. 10.1038/s41562-024-02029-w. 39468279 10.1038/s41562-024-02029-w PMC11860229

[53] Mayer SE. What money can’t buy: family income and children’s life chances. Harvard University Press, 1998.

