# Supplementary material for: Relative and combined contributions of adverse childhood experiences and self-reported child poverty to health and economic outcomes in adults – a retrospective study in a UK region
Source: BMC Public Health. 2025 Oct 15;25:3501. doi: 10.1186/s12889-025-23938-z (PMC12522892; doi:10.1186/s12889-025-23938-z)
Supplement: Supplementary file 1 — Supplementary Material 1 [file 12889_2025_23938_MOESM1_ESM.docx]

**Table A1. Questions and qualifying responses for independent variables**

|  | **Question *(response options)*** | **Qualifying response** |
| --- | --- | --- |
| **ACEs** | All ACE questions were preceded by the statement “While you were growing up, before the age of 18 years...” |  |
| *Physical abuse* | Did a parent or adult in your home ever hit, beat, kick, or physically hurt you in any way? *(never; once; more than once; prefer not to say)* | Once or more than once |
| *Verbal abuse* | Did a parent or adult in your home ever swear at you, insult you, or put you down? *(never; once; more than once; prefer not to say)* | More than once |
| *Sexual abuse* | Did someone at least 5 years older than you (including adults) ever touch you sexually, or try to make you touch them sexually? *(never; once; more than once; prefer not to say)* | Once or more than once |
| *Parental separation* | Were your parents ever separated or divorced? *(yes; no; prefer not to say)* | Yes |
| *Domestic violence* | Did your parents or adults in your home ever hit, beat, kick, or physically hurt each other in any way? *(never; once; more than once; prefer not to say)* | Once or more than once |
| *Mental illness* | Did you live with someone who was depressed, mentally ill or suicidal? *(yes; no; prefer not to say)* | Yes |
| *Alcohol abuse* | Did you live with anyone who had problems with alcohol drinking? *(yes; no; prefer not to say)* | Yes |
| *Drug abuse* | Did you live with anyone who used illegal street drugs or who abused medications? *(yes; no; prefer not to say)* | Yes |
| *Incarceration* | Did you live with anyone who served time or was sentenced to serve time in a prison or young offenders’ institution? *(yes; no; prefer not to say)* | Yes |
| **Childhood poverty** | On a scale of 1 to 10, where 1 is very poor and 10 is very wealthy, how well off would you say your household was during your childhood? *(1, very poor – 10, very wealthy; prefer not to say)* | Tertile categories: Poorest (1-4), Middle (5-6), Affluent (7-10) |
| **Chronic health condition** | Do you have any physical or mental health conditions or illnesses lasting or expected to last for 12 months or more? *(yes; no; prefer not to say)* | Yes |
| **Lower health tertile** | We would like to know how good or bad your health is today. Where 100 means the best health you can imagine and 0 means the worst health you can imagine. Please indicate how your health is today. *(0 - 100; prefer not to say)* | 0-70 |
| **Lower MWB tertile** | Short Warwick–Edinburgh Mental Wellbeing Scale (SWEMWBS©) NHS Health Scotland, University of Warwick and University of Edinburgh, 2008, all rights reserved. | Using SWEMWBS guidelines, scores for individual questions were summed and transformed into metric scores (<=23.21) |
| **Unemployed/long-term sick** | Which of the following best describes your current employment status? *(employed – full time; employed – part time; student; self-employed; unemployed; cannot work due to health reasons; retired; looking after home or family; other – please specify; prefer not to say)* | Unemployed, cannot work due to health reasons |
| **Lower income** | What is your yearly household income before any deductions such as tax? *(less than £10,000; £10,001-£20,000; £20,001-£30,000; £30,001-£40,000; £40,001-£50,000; £50,001-£60,000; £60,001+; don’t know; prefer not to say)* | less than £10,000, £10,001-£20,000 |

ACE = Adverse childhood experience, MWB = mental wellbeing.

Table A2. Comparison of regional population and sample demographics

|  |  | Regional  Population* | | Sample | |
| --- | --- | --- | --- | --- | --- |
|  |  | % | n | % | n |
| Total |  | 100 | 156766 | 100 | 5330 |
| Age | 18-24 | 11.9 | 137496 | 9.4 | 502 |
| (years) | 25-44 | 32.3 | 373147 | 32.3 | 1723 |
|  | 45-64 | 31.8 | 367449 | 33.0 | 1758 |
|  | 65+ | 24.1 | 278674 | 25.3 | 1347 |
| Sex | Male | 48.0 | 554894 | 47.5 | 2531 |
|  | Female | 52.0 | 601972 | 52.5 | 2799 |
| Ethnicity | White | 93.1 | 1304819 | 92.9 | 4954 |
|  | Other | 6.9 | 96823 | 7.1 | 376 |

*Regional population distribution: Age and Sex, Office for National Statistics, mid-2022 estimates. <https://www.ons.gov.uk/peoplepopulationandcommunity/populationandmigration/populationestimates/datasets/lowersuperoutputareamidyearpopulationestimatesnationalstatistics>. Ethnicity, Nomis, 2021 estimates of ethnic groups by Lower Super Output Area
<https://www.nomisweb.co.uk/>

**Table A3. Adjusted odds ratios for health and economic outcomes by ACEs limiting to age <65 years**

|  |  | **Health** | | | | | | **Economic** | | | | |
| --- | --- | --- | --- | --- | --- | --- | --- | --- | --- | --- | --- | --- |
|  |  | **Chronic health condition** |  | **Lower health tertile** |  | **Lower MWB tertile** |  | **Unemployed/**  **long-term sick** | | | **Lower income** | |
|  |  | AOR (95%CIs) | P | AOR (95%CIs) | P | AOR (95%CIs) | P | AOR (95%CIs) | P | AOR (95%CIs) | | P |
| **ACE** | 4+ | 3.63 (2.90-4.53) | <0.001 | 1.75 (1.41-2.18) | <0.001 | 2.25 (1.83-2.77) | <0.001 | 1.51 (1.16-1.98) | 0.003 | 2.01 (1.57-2.57) | | <0.001 |
| **count** | 2-3 | 2.23 (1.83-2.72) | <0.001 | 1.57 (1.31-1.90) | <0.001 | 2.16 (1.81-2.58) | <0.001 | 1.26 (0.98-1.61) | 0.067 | 1.70 (1.36-2.13) | | <0.001 |
|  | 1 | 1.44 (1.17-1.78) | <0.001 | 1.27 (1.05-1.54) | 0.016 | 1.28 (1.07-1.54) | 0.008 | 1.04 (0.80-1.35) | 0.791 | 1.77 (1.41-2.23) | | <0.001 |
|  | (Ref) 0 |  | <0.001 |  | <0.001 |  | <0.001 |  | 0.014 |  | | <0.001 |
| **Childhood** | (poorest) 1 | 1.41 (1.14-1.73) | 0.001 | 1.87 (1.53-2.27) | <0.001 | 1.61 (1.34-1.95) | <0.001 | 2.14 (1.64-2.80) | <0.001 | 1.83 (1.45-2.31) | | <0.001 |
| **poverty tertiles** | (mid) 2 | 0.97 (0.79-1.18) | 0.739 | 1.12 (0.93-1.34) | 0.241 | 1.22 (1.03-1.45) | 0.024 | 1.29 (0.99-1.69) | 0.056 | 1.08 (0.86-1.36) | | 0.494 |
|  | (Ref; wealthiest) 3 |  | <0.001 |  | <0.001 |  | <0.001 |  | <0.001 |  | | <0.001 |
| **Age** | 18-24 | 0.35 (0.26-0.46) | <0.001 | 0.39 (0.30-0.50) | <0.001 | 1.21 (0.97-1.49) | 0.088 | 0.61 (0.45-0.84) | 0.002 | 0.96 (0.74-1.24) | | 0.745 |
| **(years)** | 25-44 | 0.51 (0.43-0.60) | <0.001 | 0.59 (0.51-0.68) | <0.001 | 1.04 (0.90-1.20) | 0.618 | 0.55 (0.45-0.67) | <0.001 | 0.64 (0.53-0.77) | | <0.001 |
|  | (Ref) 45-64 |  | <0.001 |  | <0.001 |  | 0.233 |  | <0.001 |  | | <0.001 |
| **Sex** | Male | 0.77 (0.66-0.90) | <0.001 | 0.97 (0.84-1.11) | 0.635 | 1.06 (0.92-1.21) | 0.416 | 1.24 (1.03-1.49) | 0.023 | 0.90 (0.76-1.07) | | 0.225 |
| **Ethnicity** | White | 2.34 (1.64-3.34) | <0.001 | 1.48 (1.11-1.96) | 0.007 | 1.16 (0.91-1.49) | 0.232 | 1.55 (1.04-2.31) | 0.031 | 0.82 (0.61-1.10) | | 0.184 |

ACE = adverse childhood experience, MWB = mental wellbeing, AOR = adjusted odds ratio, CIs = confidence intervals, Ref = reference category. P values refer to difference between Ref category and other categories for each variable. P values for Ref categories identify level of significance variable contributes to model. Ref categories for sex and ethnicity are female and other than white respectively.
